# Patterns of condom use and associated factors among adult HIV positive clients in North Western Ethiopia: a comparative cross sectional study

**DOI:** 10.1186/1471-2458-12-308

**Published:** 2012-04-26

**Authors:** Estifanos Yalew, Desalegn T Zegeye, Solomon Meseret

**Affiliations:** 1 Department of Public Health, Faculty of Medical and Health Sciences, Wollega University, Nekemte, Ethiopia; 2 Department of Epidemiology and Biostatistics, School of Public Health, College of Medicine and Health Sciences, University of Gondar, Gondar, Ethiopia

## Abstract

**Background:**

The introduction of antiretroviral therapy (ART) has sharply decreased morbidity and mortality rates among HIV infected patients. Due to this, more and more people with HIV live longer and healthier lives. Yet if they practice sex without condom, those with high viral load have the potential to infect their sero-negative sexual partner or at risk of acquiring drug resistant viral strains from their sexual partner who are already infected. Hence, we aimed to assess practice of condom use and associated factors among HIV positive clients at Felege Hiwot Referral Hospital in North Western Ethiopia.

**Methods:**

Hospital based comparative cross sectional study was conducted at Felege Hiwot Referral Hospital in northwest Ethiopia. Systematic random sampling technique was used to select 466 study participants from the ART and pre ART clinic of the Hospital. A structured interview administered questionnaire first prepared in English then translated into Amharic was used to collect data. Nurses who were working in the hospital but not in the HIV clinic were recruited and trained as data collectors.

**Results:**

A total of 454 (224 respondents from ART naive and 230 ART experienced groups) were included in the study. Females constitute 151 (67.4%) and 133 (57.8%) of pre ART and ART group respectively. The ages of the participants ranged from 18 to 72 years. The average age was 31.7 years for women and 36.6 years for the men. About half of the participants (47.4% of ART group and 50.4% of the pre ART group) were sexually active. Inconsistent condom use was reported by 61(56%) ART and 50 (44.2%) of the pre ART sexually active study participants.

**Conclusions:**

The study found that those who are on ART were at lower risk of using condom inconsistently as compared to the ART naïve patients living with HIV. Therefore, these results are of high importance in order to design tailored interventions.

## Background

Antiretroviral treatment (ART) has reduced mortality and morbidity from HIV disease improving the wellbeing of many people living with HIV [1]. As a result, many HIV-infected persons are living longer and healthier lives [1,2]. Yet if they sex without condom, those with high viral load or low CD4 count before or at the initiation of ART have the potential to infect their sero-negative sexual partner or at risk of acquiring drug resistant viral strains from their sexual partner who are already infected [3-6].

The impact of growing access to ART on consistent use of condom remains an ongoing debate [3,7,8]. Studies in the developed world suggested that individuals who learn that they are HIV-infected tend to decrease their sexual risk behavior [7]. Hand full of studies were conducted in developing countries and the finding in the majority of these studies showed that access to ART has not led to significant risky sexual behavior [8-10]. However, it is still pointed out that a subset of PLWHA is still engaged in unprotected intercourse, and hence the potential risk for HIV transmission persists.

With an estimated 1.1 million people living with HIV, Ethiopia has one of the largest populations of HIV infected people in the world. Since 2005, a growing number of HIV infected Ethiopians have gained access to ART so that by 2010, 246,347 Ethiopians in need of treatment were receiving ART [11]. In Ethiopia, it remains much to be done to understand why, when, and under what conditions PLWH practice risky sexual behaviour [12]. In order to ensure that people with HIV receive high quality sexual health services, providers and policy makers must have a comprehensive understanding of the issues and challenges faced by people living with HIV.

Little is known about the practice of condom use among HIV positive patients in Ethiopia and until recently the focus of HIV prevention efforts in most countries including Ethiopia was largely on people uninfected with HIV and the sexual risk practice of HIV infected persons did not receive due attention.

## Methods

### Study setting

Hospital based comparative cross sectional study was conducted at Felege Hiwot Referral Hospital which is located 565 km north-western direction of Addis Ababa, the capital of Ethiopia. According to the hospital HIV care data base system, 16, 796 people were enrolled in the hospital in August 2010. Of these, 5136 were receiving antiretroviral drugs and 396 of them were under the pediatrics ART program. The remaining 11660 were enrolled in the pre ART care.

### Participants

The study participants were clients 18 years above and attending Outpatient HIV clinic of the hospital. Patients in the ART group were monthly provided with the standard WHO recommended first-line drugs. All patients in this group had been on ART for at least one year. ART naïve (Pre-ART) group consists of HIV-positive individuals who have known their HIV status for at least a year but who have never undergone ART. As part of the standard care they were provided with cotrimoxazole prophylaxis, management of opportunistic infections, and laboratory evaluation of HIV disease stage as clinically appropriate [13]. Patients on ART visit the HIV clinic every month and the pre ART group every three months.

EPI Info stat calc program was used to calculate the sample size. A total of 466 clients (233 from each group of ART and pre ART group respectively) were invited to participate in the study. Systematic random sampling technique was used and was adjusted based on the total number of daily visitors of the clinic in the month of August. Those patients who visited the clinic without their date of appointment due to illness or conditions, unable to communicate, mentally handicapped and seriously ill were excluded from the study.

### Measurements

The dependent variable was “Consistent condom use” (use of condom in every sexual encounter in the last three months preceding the study). The independent variables include socio demographic characteristics like age, sex, marital status, residence (urban/rural), employment status (employed/not employed); relational and behavioural factors such as type of partners (regular/non regular/commercial partners), disclosure of HIV status to partner, knowledge of sexual partner’s HIV status (yes/no), perception of stigma, substance use (yes/no) and being member of association of people living with HIV/AIDS.

Perception of stigma were assessed by using a set of 28 likert scale questions which were drawn from previous scale of stigma to address perception of stigma and HIV among PLWHA [14], and dichotomized based on the mean value of all likert scale questions.

### Data collection

A structured interview administered questionnaire first prepared in English then translated into the national language, Amharic was used to collect data. Nurses who were not working in that clinic were recruited and trained as data collectors. Nurses interviewed the participants in an isolated private room found close to the clinic. Before actual data collection pre-test was conducted with 24 clients who were not included in the main study.

### Data analysis

Data was entered and cleaned using EPI Info 2000 and analysed by SPSS V. 16. Bivariate analysis were carried out to see the association of each independent variable on the dependent variables and those who had less than 0.2 level of significance were remain in to the final models. Finally, stepwise multiple logistic regression analysis technique was carried out and p value of less than 0.05 was used as a cut off point for declaring the presence of association. Odds ratios and 95% confidence intervals were also computed.

### Ethical considerations

Ethical approval was obtained from Institutional Review Board of College of Medicine and Health Sciences, University of Gondar. Respondents were informed about the purpose, procedure, risks and benefits, and the private and confidential nature of the study. Participation was voluntary and verbal informed consent was obtained from each respondent.

## Results

### Characteristics of the sample

Of 466 respondents who were approached for participation, 12 (9 ART naive and 3 from ART experienced group) were excluded from the analysis due to incomplete data. Then a total of 224 and 230 respondents from ART naive and experienced groups, respectively, were included in the study. The characteristics of the 454 participants according to antiretroviral treatment status are summarized in Table 1. Females constitute 151 (67.4%) and 133 (57.8%) of pre ART and ART group respectively. The ages of the participants ranged from 18 to 72 years. The average age was 31.7 years for women and 36.6 years for the men. Majority of the respondents were urban resident (86.6%). A quarter of the respondents (25.1) can not read & write. Only 43% of were married at time of data collection. The majority (82.4%) had gainful Employment. Forty three percent of respondents were currently married. Approximately half (50.6%) were undergoing ARV therapy and the remainder were ARV-naive. The mean (Standard deviation) age for the ART naive and ART experienced groups were 32.5 (8.1) and 34.6 (8.3) respectively.

**Table 1:** Participants characteristics, overall and by ART status

**Variables**	**ART experienced (n = 230) N (%)**	**ART naïve (n = 224) N (%)**	**Total (n = 454) N (%)**
Sex			
Male	97 (42.2)	73 (32.6)	170 (37.4)
Female	133 (57.8)	151 (67.4)	284 (62.6)
Age			
18–25	26 (11.3)	41 (18.3)	67 (14.8)
26–35	111 (48.3)	115 (51.3)	226 (49.8)
36–45	71 (30.9)	47 (21.0)	118 (26)
> 45	19 (8.3)	15 (6.7)	34 (7.5)
Mean ±SD	34.6 ± 8.3	32.3 ± 8.1	33.5 ± 8.2
Residence			
Urban	201 (87.4)	193 (86.2)	394 (86.8)
Rural	27 (11.7)	28 (12.5)	55 (12.1)
Educational status			
Can’t read and write	58 (25.2)	56 (25)	114 (25.1)
Only read & write	21 (9.1)	25 (11.2)	46 (10.1)
Primary	58 (25.2)	55 (24.6)	113 (24.9)
Secondary	61 (26.5)	62 (27.7)	123 (27.1)
College/University	30 (13)	24 (10.7)	54 (11.9)
Marital status			
Married	100 (43.5)	99 (44.2)	199 (43.8)
Single	34 (14.8)	50 (22.3)	84 (18.5)
Widowed	48 (20.9)	38 (17.0)	86 (18.9)
Divorced	46 (20)	32 (14.3)	78 (17.2)
Employment status			
Employed	192 (83.5)	182 (81.2)	374 (82.4)
Not employed	38 (16.5)	42 (18.8)	80 (17.6)

### Patterns of condom use

A total of 222 (48.9%) respondents were sexually active (reported sexual intercourse in the prior 3 months). From this 109 (47.4%) were ART experienced and 113 (50.4%) were ART naive. There is no statistical significant difference in sexual activity between the two groups (*p* = 0.51).

All of the respondents were heterosexuals and only practiced vaginal sex. Twenty nine (6.4%) and 23 (5.1%) of the sexually active had sex with non regular and commercial sexual partner respectively. Inconsistent condom use was reported by 61(56%) ART and 50 (44.2%) of the pre ART sexually active study participants.

The common reason reported by respondents for their use of condom inconsistently were: 31 (27.9%) due to partner refusal, 24 (21.6%) due to desire of having children, 21 (18.9%) due to take away a romance in sex, 13 (11.7%) due to their partner was HIV positive, 6 (5.4%) due to not to suspicious of positive status and 4 (3.6%) was due to marital relationship.

### Factors associated with inconsistent condom use

Among those who use condom inconsistently 33 (73.3%) of them had perception of stigma of being HIV positive. Of these 50 (21.7%) and 27 (12.1%) were from ART experienced and ART naive group respectively. Perceived stigma was more reported by ART experienced groups than ART naive. This was statistically significant (*P* = 0.006).

Stepwise logistic regression was applied to identify the variables significantly associated with inconsistent condom use among both, ART experienced and ART naive groups and the results are summarized in Table 2, 3 and 4 respectively.

**Table 2:** Factors associated with inconsistent condom use among both (HIV positives) groups

**Variables**	**Consistent condom use**		**Bivariate analysis**	**Multivariate analysis**
	**Yes**	**No**	**COR [95% CI]**	**AOR [95% CI]**
Age				
< 32	45	66	1	1(ref.)
≥ 32	63	41	0.44[0.26 – 0.77]	0.41[0.21 - 0.79]
Residence				
Urban	92	90	0.51[0.22 – 1.16]	0.29[0.1 - 0.83]
Rural	18	10	1	1(ref.)
Educational status				
Can’t read & write	14	35	1	1(ref.)
Only read & write	15	11	0.29[0.11 – 0.79]	0.42[0.13 – 1.37]
Primary	32	31	0.39[0.18 – 0.86]	0.49[0.19 – 1.29]
Secondary	30	19	0.25[0.11 – 0.59]	0.18[0.06 – 0.51]
College/University	19	13	0.27[0.11 – 0.70]	0.28[0.09 – 0.88]
Member of Association				
Yes	27	15	0.49[0.24 – 0.98]	0.4[0.17 – 0.97]
No	84	96	1	1(ref.)
Presence of STI symptoms				
Yes	10	20	2.2 [0.98 – 4.94]	2.87[1.02 – 8.08]
No	100	91	1	1(ref.)
Taking ART				
Yes	61	48	1	1(ref.)
No	50	63	1.6 [0.94 – 2.72]	1.87[1.96 – 3.62]
Perceived stigma				
Stigmatized	12	33	1	1(ref.)
Non stigmatized	99	78	0.29[0.14 – 0.59]	0.35[0.14 – 0.87]
HIV transmission can occur while taking ART				
Yes	104	85	0.23[0.09 – 0.56]	0.35[0.12 – 1.02]
No	7	25	1	1(ref.)

**Table 3:** Factors associated with inconsistent condom use among ART experienced groups

**Variables**	**Consistent condom use**		**Bivariate analysis**	**Multivariate analysis**
	**Yes**	**No**	**Crude OR [95% CI]**	**AOR [95%CI]**
Sex				
Male	41	23	0.45[0.21 – 0.98]	
Female	20	25	1(ref.)	
Age				
< 32 years	22	27	1[0.19 – 0.94]	1(ref.)
≥ 32 years	38	20	0.43	0.31[0.12 – 0.84]
Residence				
Urban	48	45	0.27[0.07 – 1.01]	0.22[0.04 – 1.09]
Rural	12	3	1	1(ref.)
Marital status				
Married	51	34	1(ref.)	
Single	4	3	1.13[0.24 – 5.35]	
Widowed	3	2	1[0.16 – 6.30]	
Divorced	2	9	0.02[1.37 – 33.18]	
Member of association of PLWHA				
Yes	18	5	0.28[0.09 – 0.82]	
No	43	43	1(ref.)	
Disclosure				
Yes	59	43	0.29[0.05 – 1.57]	
No	2	5	1(ref.)	
Thinking of HIV transmission while taking ART				
Yes	58	31	0.1[0.03 – 0.37]	0.17[0.04 – 0.79]
No	3	16		1(ref.)
Use of substances				
Yes	5	10	1	1(ref.)
No	56	38	0.34[0.11 – 1.07]	0.14[0.03 – 0.68]
Perception of stigma				
Stigmatized	7	25	1	1(ref.)
Not stigmatized	4	23	0.12[0.05 – 0.32]	0.15[0.05 – 0.46]
Having knowledge of partner’s HIV status				
Yes	52	34	0.42[0.16 – 1.08]	
No	9	14	1(ref.)	

**Table 4:** Factors associated with inconsistent condom use among ART naive groups

**Variables**	**Consistent condom use**		**Bivariate analysis**	**Multivariate analysis**
	**Yes**	**No**	**COR [95% CI]**	**AOR [95%CI]**
Sex				
Male	23	19	0.51[.23 – 1.01]	
Female	27	44	1(ref.)	
Age				
< 32	23	39	1	19(ref.)
≥ 32	25	21	0.49[.23 – 1.08]	0.38[0.15 – 0.98]
Educational status				
Can’t read & write	5	22	1(ref.)	
Only read & write	8	9	0.26[.07 - 0.99]	
Primary	12	17	0.32[.09 – 1.09]	
Secondary	14	9	0.15[.04 – 0.53]	
College/University	10	6	0.14[.03 – 0.55]	
Employment status				
Employed	47	43	1	1(ref.)
Not employed	3	20	0.14[.04 - .49]	0.15[0.038 – 0.60]
Presence of STI symptom				
Yes	5	14	2.57[.86 – 7.71]	6.66[1.26 – 35.23]
No	45	49	1	1(ref.)

ART naive respondents were two times more likely to use condom inconsistently than ART experienced (AOR = 1.87 [1.96 – 3.62]). Being older in age, not perceived to be stigmatized, urban residents and a member of association of PLWHA had lower risk of practicing inconsistent condom use where as those who had sexually transmitted infections shows greater risk (Table 2).

Inconsistent condom use among ART experienced groups was lower among older age, did not use substances and those who had knowledge that HIV transmission can occur while taking ART. On the other side, those who perceived to be stigmatized had seven times more likely to use condom inconsistently than who did not (AOR = 0.15, 95% CI = 0.05 – 0.46) (Table 3). The factors associated with inconsistent condom use among ART naive groups were age, employment status and presence of sexually transmitted infections (STI) symptoms (Table 4).

## Discussion and conclusions

There is few data regarding practice of condom use among HIV positive clients in Ethiopia. This hospital based comparative cross sectional study thus provides important information regarding the practice of condom use among HIV positive clients as well as the associated risk factors.

About half the people living with HIV were sexually active (47.4% of ART experienced and 50.4% of ART naïve). This is in line with data from Thailand (56% in the previous 6 months) [15] and African countries like rural Uganda (47% at base line and 53% at follow-up) [16], Kampala (ART experienced: 55% ART naive: 45%) [17], Kenya (47.5% in the previous 12 months) [18]. However it is far less than the study done in South Africa [19] where 90% men and 81% of women reported being sexually active in the previous 3 months and India (63.2%) [20] . Hence receiving ART was not associated with increased sexual activity among Ethiopian study participants which may indirectly informs us the effectiveness of the HIV prevention program in the country.

On multivariate analysis, ART naïve respondents were more likely to use condom inconsistently than its counterpart. Hence ART was actually associated with reduced sexual risk behavior. A similar study from South Africa encompassing rural and urban clinics demonstrated that sexual risk behavior significantly decreased after ART initiation among HIV-infected South African men and women in primary care program [9]. These finding is also consistent with the results of a meta-analysis of literature from developed countries [7] . And developing countries [8] which found that the likelihood of engaging in unprotected sexual behavior was not higher among persons receiving ART compared to those not receiving ART. This could be due to the more robust HIV prevention programs and counseling among ART experienced patients compared to those who were ART-naïve.

The major reason given for not using condom consistantly were partner refusal (27.9%) and desire of having children (21.6%). This finding was in line with other Ethiopian studies [21,22]. So efforts should be strengthen on sexual health intervention focusing on the couple than the individual and monitoring of client’s attitudes and practice on their follow up period.

CD4 level and WHO stage did not show significant association with inconsistent condom use in this study. This is consistent with research done in Thailand [15]. This may be due to the selective enrolment of those patients who were on follow-up for at least one year.

In the study those who were a member of association of people living with HIV/AIDS were 40% lower at risk of using condom inconsistently than its counterpart for both ART experienced and naive groups. This result was in line with the survey done in Cameroon [23]. Hence, this finding may indicate greater access and utilization of prevention messages by members of PLWHA associations. The study also showed that those participants who thought HIV transmission can occur while taking ART were 65% lower at risk of using inconsistent condom than those who did not think so. The misconception may happen either by ignoring the ART information communicated or lack ART knowledge by HIV positive persons which made them engaged in unprotected sex. Hence, counselors are recommended to discuss the effect of ART on HIV transmission in order to avoid the misconception.

Respondents who perceived to be none stigmatized were 65% lower at risk of using condom inconsistently than its counterpart. This finding is consistent with studies done in Ethiopia and Kenya [21,24]. Hence, Counselors need the means to assist patients to cope with stigmatization and discontinue the sexual risk behavior.

Among ART experienced groups, those who did not take substances had 86% lower risk of practicing inconsistent condom use than who use substance. In a study done in Addis Ababa those who consumed alcohol were more likely to have engaged in risky sexual practice [22]. Similarly a meta analysis by Shuper and his colleagues Based on 27 studies demonstrated that any alcohol consumption, problematic drinking, and alcohol use in sexual contexts were all found to be significantly associated with unprotected sex among PLWHA [25]. This may be due to the restricted cognitive capacity stemming from alcohol consumption which causes one to focus only on impelling immediate cues [26]. So effort should be strengthen towards reduction of substance use among these particular clients.

This study has some limitations. Sexual behavior was self-reported and subject to both recall and social desirability bias. In order to minimize the recall bias we used 3 month recall period. We also tried to address social desirability bias by assigning male data collectors for male subjects and female data collectors for female subjects. Still we believe that the traditional reluctance to discuss sexual behavior may result in underreporting. Finally, being cross-sectional study it may not show the trend of sexual risk behavior over a period of undergoing ARV therapy.

In summary, though this study supports an absence of association between ART use and recent sexual intercourse we found that use of ARV therapy was associated with a decline in risky sexual behavior among sexually active individuals living with HIV. Therefore, these results are of high importance in order to design tailored interventions.

## Competing interests

The authors declare that they have no competing interests.

## Authors’ contributions

EY wrote the proposal, participated in data collection, analyzed the data and drafted the paper. DTZ and SM take part in proposal development, participated in data analysis and revised subsequent drafts of the paper. All authors read and approved the final manuscript.

## Pre-publication history

The pre-publication history for this paper can be accessed here:

http://www.biomedcentral.com/1471-2458/12/308/prepub
